# Transitions between care networks: a prospective study among older adults in the Netherlands

**DOI:** 10.1007/s10433-024-00817-x

**Published:** 2024-08-13

**Authors:** Maura K. M. Gardeniers, Martijn Huisman, Erik Jan Meijboom, Emiel O. Hoogendijk, Marjolein I. Broese van Groenou

**Affiliations:** 1 Department of Sociology, Vrije Universiteit Amsterdam, De Boelelaan, 1081, Amsterdam, The Netherlands; 2 Department of Epidemiology and Data Science, Amsterdam UMC, Location Vrije Universiteit Amsterdam, Amsterdam, The Netherlands

**Keywords:** Latent transition analysis, Formal care, Informal care

## Abstract

As health impairment increases, older adults utilize care from different types of caregivers, but little is known about changes in the composition of care networks. We mapped the transitions between different care networks to gain insight into which people develop care networks that include informal, privately and publicly paid care. We used three waves (2012–2015–2018) of the Longitudinal Aging Study Amsterdam with 1413 Dutch community-dwelling adults, aged 64–100. Network types were identified using six types of caregivers: (1) co-resident, (2) non-co-resident children, (3) other kin, (4) neighbours/friends/acquaintances, (5) publicly paid, (6) privately paid, in a latent transition analysis with mortality and moving to a care facility as missing states. Five types of care networks were identified: (1) no care, (2) privately paid, (3) mixed informal, (4) mixed publicly paid, (5) co-resident. The co-resident network was the most unstable and had a high transition rate to nursing homes. Participants from the privately paid care network often transitioned to a mixed informal network and rarely transitioned to a mixed publicly paid network. Transitions out of the no-care network were mostly to the privately paid network. The two mixed care networks were the most stable. Transitions appeared to be most triggered by deteriorating health. Transitions to institutional care were most likely in the mixed informal, mixed publicly paid and the co-resident network. Thus, these networks appear to require additional support to facilitate ageing in place.

**Supplementary Information:**

The online version contains supplementary material available at 10.1007/s10433-024-00817-x.

## Introduction

With increasing life expectancy and care policies aimed at "ageing in place", more older adults with complex health needs will require care at home for a considerable number of years (Böcker et al. 2017; WHO 2015). As the need for care increases, a network usually develops around the person in need of care, consisting of different types of caregivers. The caregivers can be informal caregivers from the social network (e.g. spouses, relatives, friends and neighbours or volunteers) or formal caregivers (professionals) who are either publicly or privately paid. The ratio between the number of informal, publicly and privately paid caregivers can vary over the course of the care trajectory.

Previous research on care arrangements can be broadly divided into two traditions. The first tradition examines care arrangements longitudinally and divides caregivers into the rather broad categories of formal, informal or a mixture of formal and informal care (Bonsang 2009; Geerts et al. 2012; Kjaer & Siren 2019; Tennstedt et al 1996). These studies show that most older adults receive a mixed care arrangement in the later stages of their care trajectory.

The other tradition uses a network approach (Wenger 1991, 1997; Jacobs et al. 2018; Keating et al. 2003; Keating and Dosman 2009) and distinguishes different constellations of care arrangements in more detail, usually at a specific point in time. This network approach has the advantage that it makes it possible to zoom in on these mixed care arrangements and generate ideal–typical networks in which the simultaneous occurrence of formal and informal care is made visible. These ideal–typical networks generally have different needs; for example, in networks in which formal and informal caregivers are present, communication between the caregivers should be facilitated (Jacobs et al. 2016). Networks involving only informal caregivers can be facilitated by various ways of supporting caregivers. If these needs can be linked to demographic characteristics, it will be easier for policy makers to promote the appropriate forms of support when needed.

Previous studies on care networks (CNs) have identified between four and eight types of CNs (Bijnsdorp et al 2018; Broese van Groenou et al 2016; Fret et al 2017; Jacobs et al 2018; Keating & Dosman 2009; Savla et al 2016), which differ in terms of size and the ratio of formal and informal caregivers. These network types generally included: a co-resident or spousal network, an informal network without a co-resident caregiver, a network consisting mainly of publicly paid caregivers, a CN with only (one) privately paid caregiver(s), and mixed networks containing both informal and formal caregivers in varying proportions (e.g. largely informal or largely formal). In general, the differences between these types of CNs are related to the individual determinants of care use: the need for care (health impairment), the willingness to use care (related to gender, age and education) and the ability to access certain types of care (e.g. the presence of a spouse for spousal care) (Jacobs et al. 2018). These cross-sectional findings suggest that transitions in CNs are largely determined by changes in health and/or social resources. Cross-sectional findings can shed light on the relationships between determinants and transitions, as they shed light on what conditions need to be in place for a certain type of CN to form. We know from previous studies that the presence of a partner is highly associated with the co-residential network (Jacobs et al. 2018; Keating and Dosman 2009), that higher levels of education are more common in privately paid CNs (Bijnsdorp et al. 2018; Broese van Groenou 2020; Jacobs et al. 2018) and that older age and more severe health impairment are found in the publicly paid network due to eligibility requirements for publicly paid care.

From a policy perspective, it is important to gain insights into which CNs are likely to include publicly paid carers over time. However, to date, neither transitions in CNs nor the associated determinants have been investigated, as all studies on CNs used a cross-sectional design (e.g. Jacobs et al. 2018; Keating and Dosman 2009). In addition, the Dutch healthcare system was reformed in 2015, with a shift from institutional care to facilitating ageing in place. The 2015 reform entailed decentralization to municipalities, which were expected to provide this type of care more efficiently. As a result, funding for the provision of long-term home care was significantly reduced, by 35% compared to the funding that central government had previously spent on it (Alders and Schut 2019). Eligibility criteria for institutional care were raised and initially payment for care was income-related, with higher incomes paying more for care (Alders and Schut 2019). In the run-up to the reform, politicians were quick to suggest that citizens should make more use of informal care from their own social network (Ministry of Health, Welfare and Sport 2013).

Given the transitions in long-term care policies, more insight is needed. A look at the welfare state also sheds light on what individual level factors might be associated with transitions in certain CNs. Before the reform, the Dutch long-term care system for older adults had a high degree of defamilization (Saraceno and Keck 2010), so that responsibility for care lay primarily with the state. However, the transition involved a shift to a system with a higher degree of familialization and marketization, as it was pointed out that older adults are increasingly dependent on informal and privately paid care. Considering this, one would expect fewer transitions to publicly paid care after 2015. At the same time, the increased eligibility criteria for institutional care following the reform may have led to more people making use of publicly paid care at home.

In this study, we examine CNs using a prospective design covering an observation period of (2 × 3) 6 years. We will describe what types of CNs exist, what transitions between types of CNs take place, which CNs are most stable and what health and socioeconomic characteristics of care recipients are associated with these CNs.

## Materials and methods

### Study sample and design

We used data from the Longitudinal Aging Study Amsterdam (LASA), an ongoing population-based longitudinal study of older adults (aged 55+) in the Netherlands (Hoogendijk et al. 2016, 2020). The baseline sample was stratified by age and gender from urban and rural regions of the Netherlands in 1992/93 and comprised 3107 respondents aged 55–84 years. In 2002 and 2012, two additional cohorts of 55–64 year olds were added with 1002 and 1023 respondents, respectively, from the same sample but later birth cohorts. The baseline cooperation rates were 62% for the first and second cohorts and 63% for the third cohort. Data were collected through face-to-face interviews and self-completed questionnaires. If respondents refused or were unable to participate in the standard interview, they (or a proxy) were interviewed by telephone or in an abbreviated face-to-face interview. Additional measurement waves were conducted every 3 years. The present study included all participants living in a community at baseline who were 65 years or older in the 2012/13 wave (*N* = 1413), and covered the three measurement waves 2012/13, 2015/16 and 2018/19. The survey was administered in person to 871, 842 and 742 respondents in the first, second and third waves, by telephone to 34, 43 and 86 respondents, and by proxy to 17, 37 and 92 respondents.

### Dependent variables

#### Care network

Respondents were asked whether and from whom they receive help in the following areas: personal care, home care, nursing care, transportation and administrative tasks. We categorized six types of caregivers: (1) co-resident (spouse, co-resident children and/or others), (2) non-co-resident children, (3) other relatives, (4) neighbours/friends/acquaintances, (5) publicly paid (community nurse, help at home), (6) privately paid (private help or in-home staff). For the follow-ups, we included two absorbing states: (1) moved to a care facility, (2) deceased, for which we used data from the municipal register (GBA).

### Independent variables

We selected potential determinants based on the three dimensions of Andersen and Newman's (2005) behavioural model of health service use: need, predisposing and enabling factors. Need factors include all factors that increase the need for care, such as chronic illness, functional limitations and cognitive decline. Predisposing factors are factors such as demographic characteristics and attitudes towards care that indicate a willingness to ask for help. Enabling factors facilitate the use of care, e.g. the presence of potential caregivers in the social network.

#### Need variables

Functional limitations are measured as the respondents’ ability to perform the following six activities of daily living: (1) dress or undress themselves, (2) get up from a chair or sit down, (3) cut their own toenails, (4) use their own or public transportation, (5) climb stairs and (6) walk outside for five minutes without resting. Responses were summed and ranged from 6 to 30, with higher scores indicating a higher level of functioning (Pluijm et al. 2005).

Cognitive functioning was measured using a shortened version of the Mini-Mental State Examination (sMMSE) (Folstein et al. 1975; Tombaugh and McIntyre 1992). The scale ranges from 0 to 16, with higher scores indicating better cognitive functioning. For respondents who were unable to complete this questionnaire, cognitive functioning was assessed in a proxy interview and measured using a shortened version of the IQCODE (Jorm and Korten 1988). To make the measures of cognition comparable over time, IQCODE scores were converted to sMMSE scores using cut-off points based on an earlier LASA study by Comijs et al. (2004).

Chronic diseases were recorded as the sum of seven types of chronic diseases (range 0–7): lung disease, heart disease, arterial disease, diabetes, cardiovascular accidents, rheumatic diseases and cancer.

The change scores for these variables were calculated by subtracting the previous wave's score from the current wave's score, resulting in one baseline score and two change scores per variable.

#### Predisposing factors

These include age at baseline (in years), sex (1 = female) and education level, which was categorized into three groups: low (elementary school), medium (secondary school or lower vocational education) and high (higher vocational education or higher).

#### Enabling factors

Partner status with three categories (partner/no partner/lost partner compared to previous wave) was assessed at each wave to differentiate between the effects of losing a partner and not having a partner.

We did not include income as a determinant because in our dataset the income variable contained a lot of missing values. Moreover, a previous study on LASA data showed that income often has similar associations as education (Abbing et al. 2022). We did not include information on working lives, as the Dutch pension age was 65 at the time of our study, so almost all of our respondents were retired. We did not include information on number of children since the proportion of people who were childless was rather stable between cohorts.

### Method of data analysis

**Fig. 1:**
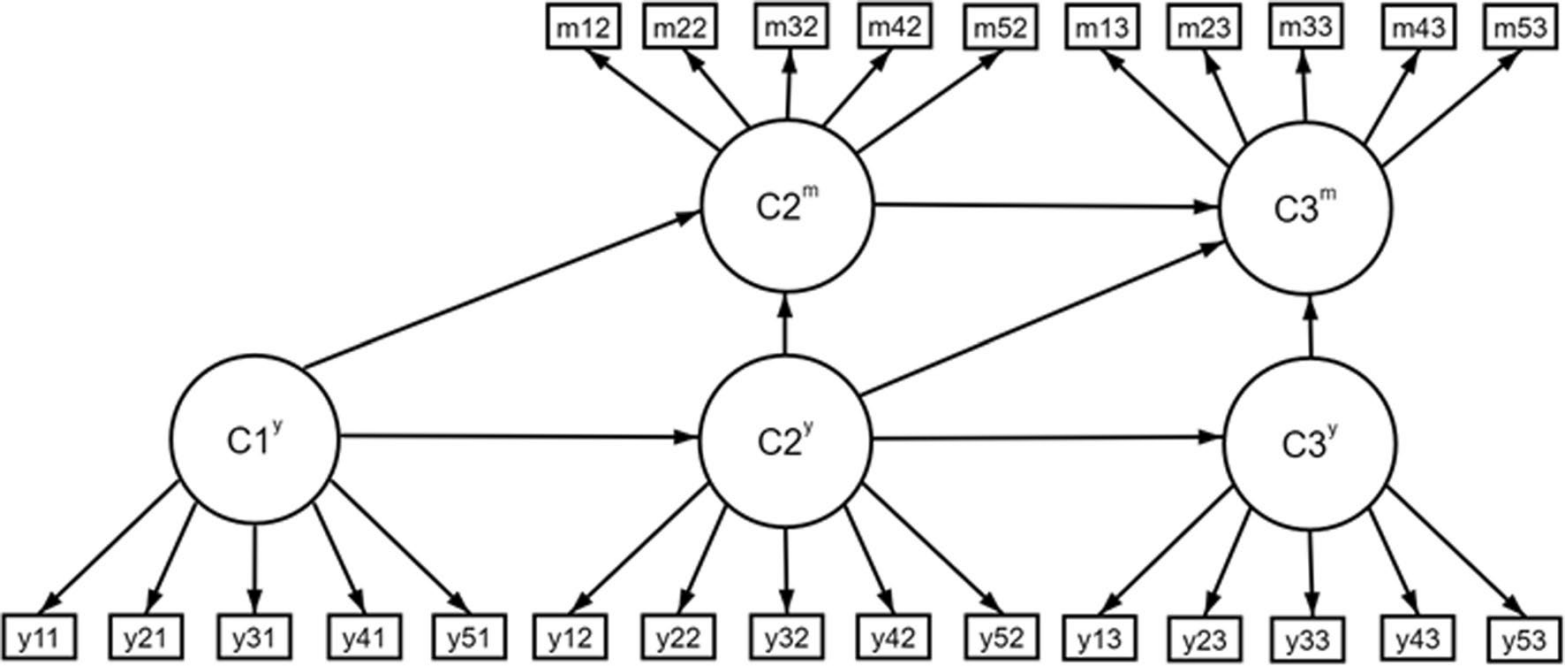
Conceptual view of the parallel-process missing-not-at-random (MNAR-PP) latent transition analysis (LTA). Shown for time = 3, and number of groups *J* = 5. *Notes* Circles represent latent classifications variables, arrows represent regression paths, squares represent measured variable indicators of the latent classification variables (Sterba 2016)

We calculated descriptive statistics using SPSS 27. We used a method that allowed for co-modelling of non-random dropouts such as death and moving to a care facility. This is important in longitudinal studies of older adults, as there is a strong association between high care needs and dropping out due to mortality or moving to a care facility. The exclusion of these departed respondents likely leads to the exclusion of respondents with high care needs. Thus, this co-modelling led to more accurate estimates of the groups that are typically of most interest to policy makers. We conducted a latent transition analysis (LTA) using MPLUS 8.6 (Muthén and Muthén  2012), together with the extension provided by Sterba (2016) in the form of a late-state-dependent nonignorable missingness LTA model (MNAR-PP LTA), which allows for co-modelling of censoring due to death and moving to a nursing facility. LTA is a longitudinal form of latent class analysis (LCA) in which the classes (called states in LTA) consist of the CNs that form around an older adult. In this model (see Fig. 1 for the conceptual representation), the probability of moving into one of the identified latent states or missing states is assessed at each time point.

Using the paper by Sterba (2016) as a guide, we tested several LTA models with different numbers of states for the outcome variables and missingness. We chose the appropriate number of states based on the lowest Bayesian information criterion (BIC), lowest log-likelihood, entropy and qualitative assessment of an additional state that is structurally different from the states identified in the *N* − 1 state solution, favouring the simplest number of latent states.

Because of power issues, we did not model the predictors and provided the descriptives instead (Table 1).

**Table 1:** Descriptive statistics of sample total and CN types at each wave

	Total	At 2012/2013 (T1) (*N* = 1413)					At 2015/2016 (T2) (*N* = 1182)				
		No care	Private	Mixed informal	Mixed public	Co-residential	No care	Private	Mixed informal	Mixed Public	Co-residential
*N*	1413	837	156	108	213	99	629	188	120	180	65
% of total	100	59.24	11.04	7.64	15.07	7.00	53.21	15.91	10.15	15.23	5.50
Female %	0.56	0.48	0.63	0.7	0.72	0.62	0.48	0.61	0.79	0.65	0.68
Age in years	74.92 (0.76)	71.56 (5.61)	76.89 (7.09)	81.76 (7.76)	82.38 (6.25)	76.73 (7.23)	70.29 (4.65)	75.13 (6.24)	78.1 (6.53)	79.61 (6.37)	75.18 (6.59)
Partner at T1%	0.67	0.79	0.60	0.27	0.34	0.92	0.81	0.68	0.49	0.43	0.95
Partner at T2%	0.63	0.74	0.50	0.13	0.29	0.94	0.79	0.60	0.30	0.29	0.95
Partner at T3%	0.60	0.70	0.49	0.11	0.18	0.79	0.75	0.54	0.24	0.18	0.84
Moved to a care facility at T2%	0.04	0.01	0.03	0.16	0.14	0.08					
Moved to a care facility at T3%	0.05	0.03	0.06	0.11	0.19	0.15	0.03	0.06	0.11	0.19	0.15
ADL-score at T1^a^	25.59 (5.82)	27.7 (4.46)	26.13 (5.08)	22.11 (6.11)	21.15 (5.90)	20.15 (6.05)	27.97 (4.36)	26.66 (5.06)	23.41 (4.28)	22.62 (6.18)	22.89 (5.90)
ADL-score at T2	25.11 (6.15)	27.06 (4.83)	25.17 (5.73)	20.65 (6.46)	19.48 (6.04)	18.57 (6.85)	27.82 (4.13)	26.43 (4.77)	25.35 (5.11)	20.84 (5.77)	19.45 (6.73)
ADL-score at T3	24.12 (6.48)	25.62 (5.77)	23.75 (6.35)	19.66 (6.75)	18.5 (5.45)	18.05 (7.06)	26.3 (5.46)	24.66 (5.88)	22.92 (5.95)	19.35 (5.78)	18.66 (6.51)
sMMSE-score at T1^b^	14.82 (1.69)	15.15 (1.24)	15.17 (1.32)	14.36 (1.94)	14.05 (2.13)	13.63 (2.68)	15.19 (1.19)	15.23 (1.19)	20.78 (6.28)	14.68 (1.71)	14.09 (2.44)
sMMSE-score at T2	14.44 (2.23)	14.94 (1.53)	14.38 (2.17)	13.57 (2.81)	12.83 (3.21)	12.85 (3.46)	15.04 (1.26)	14.91 (1.51)	15.08 (1.22)	13.60 (2.68)	13.45 (3.19)
sMMSE-score at T3	14.29 (2.51)	14.71 (1.91)	14.37 (2.36)	13.05 (3.61)	12.42 (3.60)	12.62 (3.87)	14.89 (1.57)	14.59 (1.90)	14.41 (1.93)	12.97 (3.43)	12.87 (3.90)
*N* Chronic diseases at T1^c^	1.49 (1.16)	1.25 (1.04)	1.55 (1.09)	1.71 (1.22)	2.11 (1.22)	1.81 (1.36)	1.18 (1.00)	1.44 (1.09)	13.65 (2.75)	1.86 (1.15)	1.74 (1.14)
*N* Chronic diseases at T2	1.53 (1.13)	1.41 (1.07)	1.55 (1.09)	1.81 (1.16)	1.99 (1.29)	1.64 (1.23)	1.35 (1.043)	1.49 (1.07)	1.59 (1.16)	2.02 (1.23)	1.75 (1.14)
*N* Chronic diseases at T3	1.63 (1.18)	1.54 (1.16)	1.67 (1.12)	1.85 (1.22)	2.12 (1.22)	1.76 (1.18)	1.48 (1.15)	1.65 (1.13)	1.68 (1.19)	2.00 (0.46)	1.82 (1.11)
Deceased at T2%	0.13	0.05	0.17	0.24	0.29	0.32					
Deceased at T3%	0.24	0.11	0.28	0.42	0.51	0.45	0.03	0.07	1.86	2.12	1.77

	Total	At 2018/2019 (T3) (*N* = 1027)				
		No care	Private	Mixed informal	Mixed Public	Co-residential
*N*	1413	484	175	172	140	56
% of total	100	47.13	17.04	16.75	13.63	5.45
Female %	0.56	0.44	0.59	0.78	0.67	0.68
Age in years	74.92 (0.76)	69.50 (4.11)	73.16 (5.79)	75.75 (5.96)	77.37 (6.51)	74.15 (5.85)
Partner at T1%	0.67	0.83	0.74	0.58	0.52	0.91
Partner at T2%	0.63	0.82	0.69	0.44	0.43	0.91
Partner at T3%	0.60	0.81	0.65	0.32	0.26	0.94
Moved to a care facility at T2%	0.04					
Moved to a care facility at T3%	0.05					
ADL-score at T1^a^	25.59 (5.82)	28.06 (4.31)	26.72 (5.06)	26.43 (5.83)	23.76 (5.97)	23.73 (6.03)
ADL-score at T2	25.11 (6.15)	28.14 (3.96)	26.82 (5.17)	24.69 (5.41)	22.92 (5.76)	21.98 (6.87)
ADL-score at T3	24.12 (6.48)	26.96 (5.17)	25.5 (5.60)	22.34 (6.15)	20.38 (5.82)	20.36 (6.08)
sMMSE-score at T1^b^	14.82 (1.69)	15.23 (1.14)	15.25 (1.12)	15.12 (1.21)	14.93 (1.45)	14.34 (2.07)
sMMSE-score at T2	14.44 (2.23)	15.11 (1.16)	15.07 (1.49)	14.87 (1.34)	14.41 (1.82)	13.70 (2.76)
sMMSE-score at T3	14.29 (2.51)	15.05 (1.28)	14.88 (1.83)	14.2 (2.09)	13.79 (2.52)	12.89 (3.62)
*N* Chronic diseases at T1^c^	1.49 (1.16)	1.09 (0.97)	1.39 (1.00)	1.55 (1.12)	1.76 (1.17)	1.5 (1.03)
*N* Chronic diseases at T2	1.53 (1.13)	1.25 (1.03)	1.42 (1.01)	1.65 (1.17)	1.88 (1.17)	1.71 (0.92)
*N* Chronic diseases at T3	1.63 (1.18)	1.4 (1.11)	1.67 (1.17)	1.76 (1.14)	2.05 (1.21)	1.9 (1.09)
Deceased at T2%	0.13					
Deceased at T3%	0.24					

^a^Activities of daily living, 6–30, with 30 indicating no problems in performing activities of daily living

^b^Short mini mental state examination, 0–16, with 16 indicating no cognitive decline

^c^Number of chronic diseases, 0–7

## Results

In the first wave, the average age was 74.9 years, 56% were female, and 67% had a partner. In the second wave, 4% had moved into a nursing home and 13% had died. In the third wave, 5% of all participants had moved into a care home and 24% had died.

The adjustment statistics of our LTA models were inconclusive. A five-state solution with two states for missingness in wave 2 and wave 3 fit the data best, for several reasons: The BIC between models did not differ substantially, the entropy was lowest for this number, and the five-state solution identified the co-residential network as one of the networks, i.e. a network we expected based on previous studies. The details of this procedure can be found in Supplementary File 1.

**Fig. 2:**
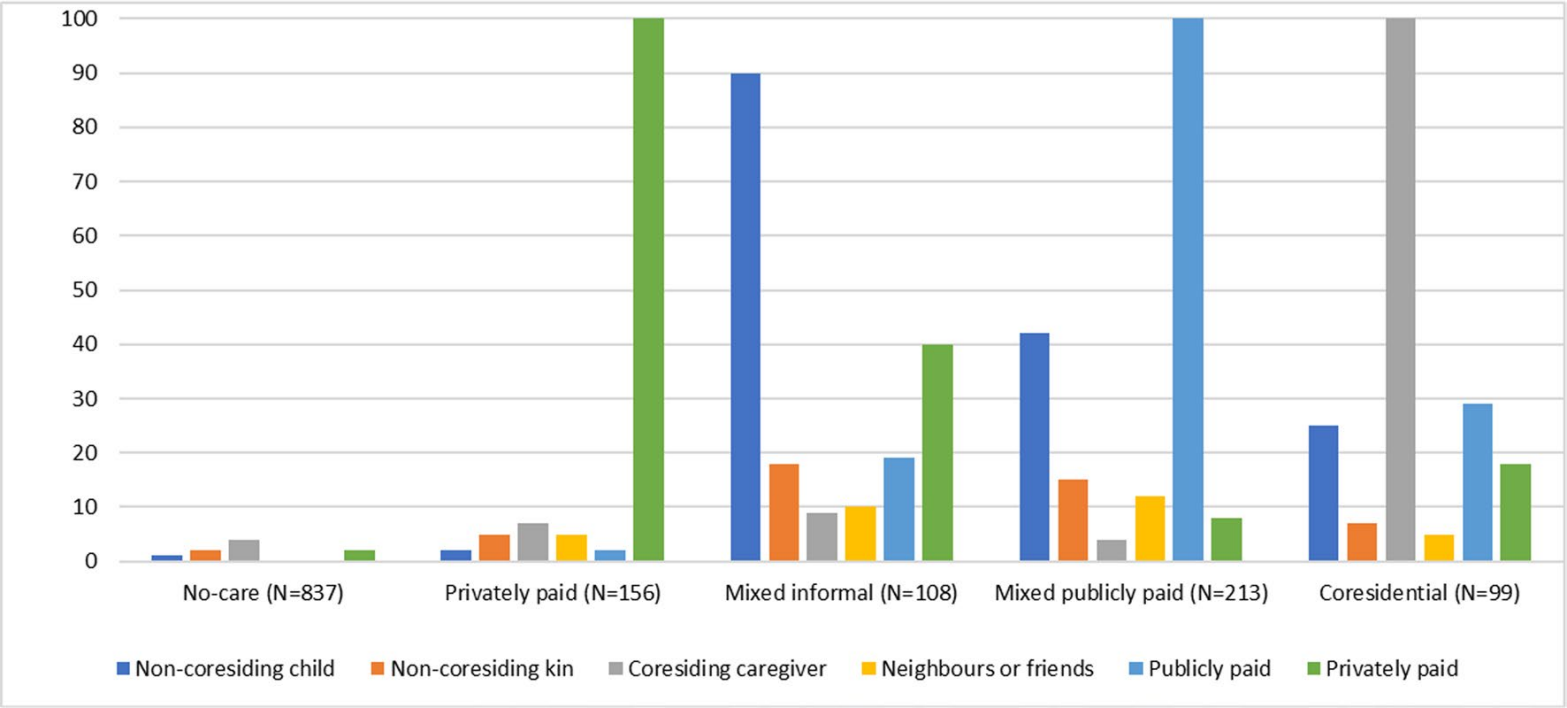
Percentages of types of caregivers in the five identified care network states, among 1413 Dutch older adults aged 65 and older. Care network types were assumed to be measurement invariant, and were assessed at T1 (2012/13), and held constant for 2015/16 and 2018/19

**Fig. 3:**
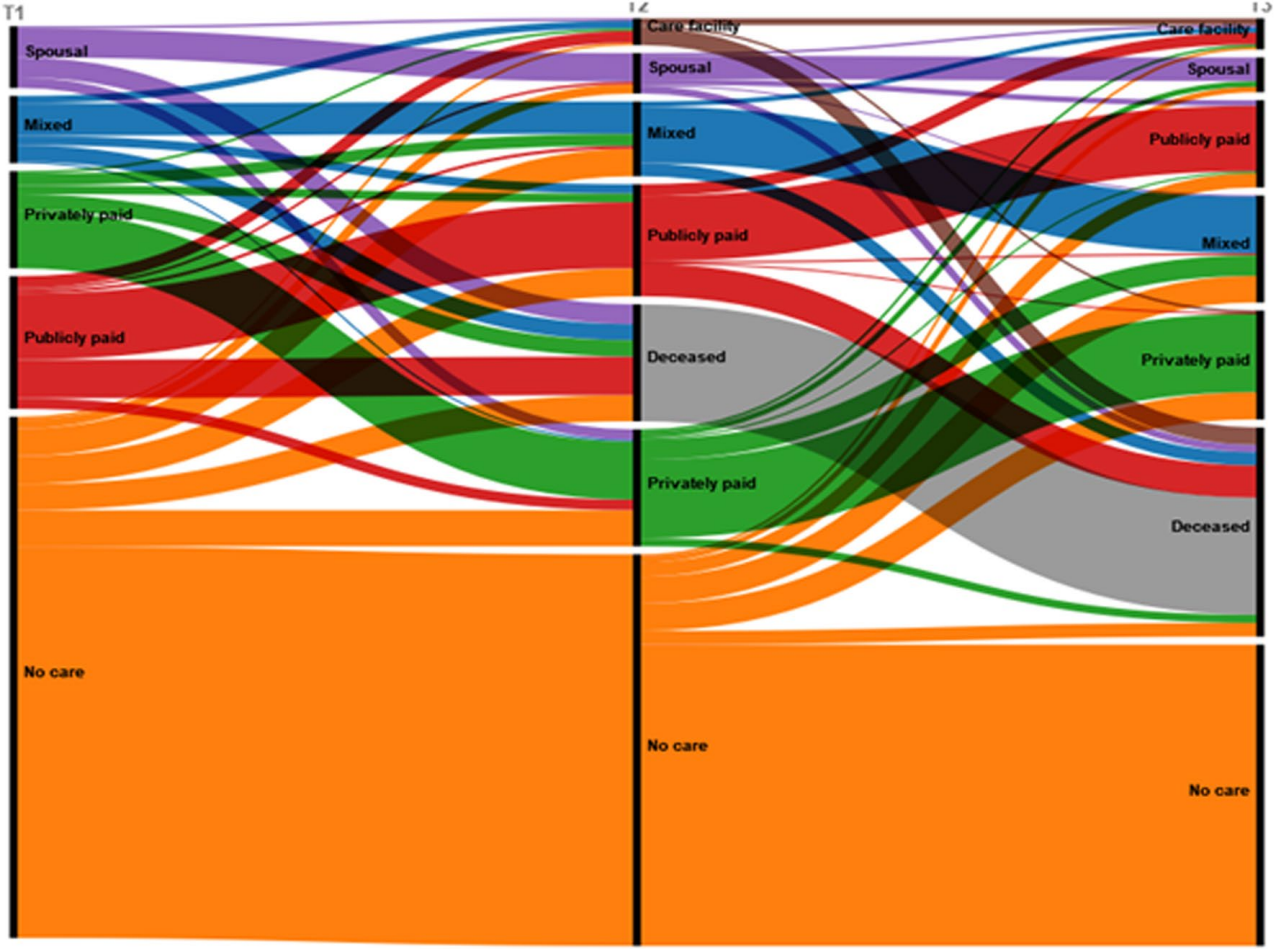
Transitions between the five CN types, from 2012/13 (T1), to 2015/16 (T2), and from 2015/16 to 2018/19 (T3). Colours represent the possible states, and the sizes are the proportions of participant in these states. The states are: the spousal or co-resident CN, mixed or mixed informal CN, privately paid or privately paid CN, publicly paid or mixed publicly paid CN, no care or no-CN

Figure 2 shows the item response probabilities for each response category within the five-state solution. It is assumed that the types of CNs are similar across all waves. Table 2 also shows the overall probabilities for each status at each time point (columns) and the transition probabilities given latent status membership at each time point (rows). Figure 3 shows these transition probabilities in an alluvial plot.

**Table 2:** Transition probabilities between the five CN types, identified among 1413 Dutch older adults aged 65 and older. Rows show CN types at time 1 and time 2, and columns show CN types at time 2 and time 3

	No care	Private	Mixed informal	Mixed public	Co-residential	Care facility	Deceased
*CN at time 1 (rows), CN at time 2 (columns)*							
No care	.751	.069	.053	.054	.018	.005	.050
Private	.000	.596	.122	.090	.000	.026	.167
Mixed informal	.000	.028	.481	.139	.000	.111	.241
Mixed public	.000	.080	.023	.498	.019	.089	.291
Co-residential	.000	.172	.000	.000	.465	.040	.323
*CN at time 2 (rows), CN at time 3 (columns)*							
No care	.717	.065	.064	.034	.013	.009	.098
Private	.000	.426	.108	.010	.030	.030	.397
Mixed informal	.000	.000	.758	.000	.000	.067	.175
Mixed public	.000	.004	.012	.423	.000	.093	.468
Co-residential	.000	.015	.031	.138	.569	.062	.185

### No care

Respondents in the first state "no-CN" (59%_T1_) received hardly any care. As shown in Table 1, respondents in this state were the youngest, with relatively high levels of functioning and few chronic diseases. There were slightly more men in this network (52%), and respondents had a higher level of education than the overall sample, 79% had a partner.

Most of these respondents remained in this network type at time 2 (75%), and 5% died. Around 20% moved to another network type, with the largest proportions transitioning to the privately paid (7%), mixed informal CN (5%) and mixed publicly paid CN (5%). At time 3, these transition rates were similar. Participants who transitioned to another network type experienced function losses: ADL-scores decreased on average by − 2.7, sMMSE decreased by − 0.5 and chronic conditions increased by 1.64, compared with − 0.5 ADL, − 0.2 sMMSE and 1.35 chronic conditions among those who remained (calculations not shown). Of those who transitioned 13% lost a partner, compared to 3% of those who remained in the group.

### Privately paid

These participants all received privately paid care (11%_T1_) and generally no other forms of care. There were relatively many men in this group (37%). They had an average age, were less likely to have a partner and had a medium or high level of education, with high levels of functioning. At Time 2, most of these respondents remained in the privately paid CN (59.6%), 16.7% died and about 25% transitioned: 12% to a mixed informal, 9% to a mixed publicly paid and 3% to a nursing home. About 11% transitioned to mixed informal CN, indicating that informal caregivers supplemented the care already provided. The transition to a mixed informal CN was associated with a loss of function, as the average decline among participants that made this transition was − 4.3 ADL-score and − 4.3 sMMSE points. Of those who transitioned at T2, 8% lost a partner compared to 6% of those who remained in the group. Mortality was high in this group at time 3: 40%.

### Mixed informal

The mixed informal CN (7.6%_T1_) shows a wide variety of caregivers: 90% received help from non-resident children, 40% from a privately paid caregiver, and between 20 and 10% received help from non-resident kin, a co-resident caregiver, neighbours or friends, and/or a publicly paid caregiver. The care recipients were relatively old, women, with no partner, a lower level of education and moderately good physical and cognitive functioning.

At T2, 48.1% remained in this network and mortality was moderately high (24%), while 13.9% transitioned to a mixed publicly paid CN and 11% to a nursing home. This was likely due to severe loss of function, as the mean decline in ADL and sMMSE for these participants were 10 and 2.8 points, respectively. Of all participants who transitioned, 9% lost their partner, compared to 12% of participants who remained in this network. In the last wave, 76% of participants remained in the network, 17.5% died and 7% moved to a care facility. This network appears to be relatively stable, although it is not known whether more or different informal caregivers joined the CN. Of the participants who made a transition, 9% lost a partner at T2 and 0% at T3.

### Mixed publicly paid

In the mixed publicly paid CN (15%_T1_), 100% of older adults were cared for by a publicly paid caregiver. They also received help from non-resident children (43%) and from co-resident caregivers (5%), neighbours or friends (10%), privately paid caregivers (8%) and non-resident kin (15%). Respondents in these network were: women, older, single, with a low level of education and poor health (ADL = 21.15, sMMSE = 14.05, 2.11 chronic diseases).

Half of these participants remained in this state at time 2 (50%), and 29% died. Almost 9% moved to a nursing facility and 8% to a privately paid CN. The participants who moved to a nursing facility had severe functional decline (mean − 5.7 ADL, − 2.6 sMMSE), while the participants who moved to a privately paid CN had mild functional decline (mean − 2.6 ADL, − 1.2 sMMSE). At time 3, participants were most likely to die (47%), and 42% remained in the network. The proportion of participants who moved to a care facility was highest in this group: 9%.

### Co-resident

In the co-resident network (7%_T1_), all respondents received help from a co-resident partner or kin. Some also received care from non-co-resident children (25%), as well as from publicly paid (28%) or privately paid (20%) caregivers. The network consisted of 62% women who were not very old (76.7 years), often had a partner (92%) and had a lower level of education. The participants had a low level of physical and mental functioning (ADL = 20.15, sMMSE = 13.6). Most participants remained at time 2 (46.5%), relatively, many died (32.3%), and 17.2% moved into privately paid CN. Of those who moved to privately paid CN, 24% lost a spouse. Only 4% moved to a nursing home. At T3, 56.9% remained and 19% died. 13.8% transitioned to the mixed publicly paid CN. Looking at this group in more detail, we find that none of them lost a partner, but all of them experienced a deterioration in their functioning (− 3.6 ADL, − 0.6 sMMSE). At T3, 6.2% moved to a nursing home.

## Discussion

To our knowledge, this study was the first to use CN typology among older adults in a longitudinal perspective. We identified the following five types of CNs: the no-CN (59%_T1_), the privately paid CN (11%_T1_), the mixed informal CN (8%_T1_), the mixed publicly paid CN (15%_T1_) and the co-resident network (7%_T1_). By accounting for non-random attrition, we were able to include meaningful information, such as transition rates to care facilities, and include deceased participants who were likely to have the highest care needs. The mixed publicly paid CN and the mixed informal CN had relatively high numbers of deceased respondents and were relatively stable. This seems to indicate that these networks are only present in the late stages of the care trajectory. In addition, the low transition rates between these two networks may indicate the existence of two separate and distinct care trajectories, with either the mixed publicly paid CN or the mixed informal CN serving as the end point.

Both network types contained more older women with low levels of education and low functioning. Differences between them could be due to social or psychological resources (Jacobs et al. 2018), which were not considered in this study. Mortality was lower in the privately paid CN and the no-CN, but also not negligibly low. Although most participants in the no-CN and privately paid CN were likely to be at the beginning of their care trajectory, some of them died before ever using any form of publicly paid care. It is likely that these participants suffered a sudden unexpected death, which is related to the fact that more men are represented in these groups and men have a higher risk of sudden death (Lewis et al. 2016; van Campen et al. 2013.

As the Dutch care reform mainly entailed overall budget cuts for publicly paid care and reduced availability of institutional care, we assume that other types of care, including informal and privately paid care, became more prominent after the care reform. After the care reform, a significantly lower percentage of older adults transitioned into a publicly paid network or care facility. Our results show what has changed over time, but as our study design did not include comparisons with other cohorts or time periods, it is possible that these results are due to changes caused by other processes.

Looking at transitions between different types of CNs and neglecting transitions due to death, the more unstable networks (for survivors) were the privately paid CN (18%_T1_) and the no-CN (19%_T1_). In these two networks, the need for care was rather low to begin with. Their instability can therefore be attributed to an increasing need for care. Both network types contained more men, participants who were relatively young, had a medium or high level of education, and had high levels of cognitive and physical functioning, all of which are indicators of low care needs (Keating et al. 2003). Privately paid care is usually a (preferred) substitute for publicly paid care (Geerts et al. 2012; Swinkels et al. 2016). Therefore, previous studies also report that a medium or high level of education, which is associated with higher affluence, contributes to the use of privately paid care (Kemper 1992; Pinquart and Sörensen 2002).

The co-resident network (25%_T1_) was the least stable network and fell between the other four network types in terms of characteristics. It contained more men and younger participants, but also more participants with low and medium levels of education than the network without care and privately paid CN. The need for care was high, and the levels of functioning were equal to or worse than that of the mixed publicly paid CN. This high need for care probably led to the higher transition rates. These high transition rates can probably also be explained by the fact that the co-resident network is highly dependent on a single caregiver, who also often tends to become frail or prefrail (Potier et al. 2018). As our descriptive study showed, the main reason for leaving the co-resident network was not the loss of a spouse, but was deteriorating health. It should be noted that we only considered whether the spouse was still alive or not, so this may not capture the full picture, as spouses can also become ill and no longer be able to act as a potential caregiver.

Our results are highly dependent on the structure of the Dutch healthcare system and the political context. As the healthcare systems in various countries are very different (Geerts and Bosch 2012), we cannot make any statements about the generalizability of the results. Our study took place in a policy context in which the eligibility criteria for transition to a care facility were very high (round-the-clock care needs). If these thresholds had been lower, the rate of transition to a care facility would very likely have been significantly higher (Alders et al. 2019). This notion is also supported by the fact that the level and type of care utilization in the Netherlands differ between decades (Abbing et al. 2021). In countries where the healthcare system is organized differently and where people have different norms regarding the use of care services, CNs are likely to look different from those identified in this study. The formation of CNs is also influenced by living arrangements, for example, Velkoff (2001) reports that women in industrialized countries are more likely to live alone than men. This is consistent with our mixed publicly paid CN and mixed informal CN containing more women, and the spousal CN containing more men. However, in non-industrialized countries, older adults tend to live with children (Velkoff 2001), so the relative proportions of different types of CNs are likely to differ significantly from those in industrialized countries. In the Dutch context in particular, the proportion of older adults living alone or with a spouse is relatively high (United Nations, Department of Economic and Social Affairs 2017). The proportion of older adults living with children is the lowest compared to other European countries (5%) (United Nations, Department of Economic and Social Affairs 2017). It is therefore likely that informal CNs involving children are more prevalent in Mediterranean countries, where around 30% of older adults live with children (United Nations, Department of Economic and Social Affairs 2017). Similarly, it is likely that studies that have measured other types of care (e.g. without help with administrative tasks or transportation) also find different CNs with lower levels of informal care. These differences in both the data and the socio-cultural context could explain why a study looking at CNs in Belgium found more people with informal CNs (Fret et al. 2017). It could also explain that a Canadian study found an informal network consisting mainly of non-kin (Savla et al. 2016), while we did not identify such a network.

Our results shed light on which CNs fit into a financially sustainable care system in which care for older adults includes low public expenditure alongside the use of informal and privately paid care. With this in mind, the no-CN, privately paid CN, co-resident CN and mixed informal CN are likely to be the most financially sustainable from a policy perspective when less publicly paid care is available. For these people, using only or mainly informal and/or privately paid care appears to be sufficient, even for those with high care needs. Some of them may move into CNs with publicly paid care in the future, as we only followed participants for 6 years. However, a sizable group never uses publicly funded care in their lifetime: these are the participants from the privately paid CN and the no-CN that died.

In terms of a financially sustainable care system, the policy aims for ageing in place. This enables older adults to maintain social connections, a sense of independence and identity (Iecovich 2014; Kendig et al 2017). Also, ageing in place is a policy goal because it is less costly than institutional care (Maarse and Jeurissen 2016). Transitions to a residential care facility appear to occur from all CNs for those experiencing the most severe functional decline, which is consistent with the eligibility criteria for institutional care. These transitions occurred mostly from the networks with a high care need: the mixed informal CN, the mixed publicly paid CN and the co-resident CN. Consistent with other studies, the likelihood of transitioning to a nursing home was higher when informal care was provided by children than by spouses (Witvorapong 2011). Nursing homes, especially with increased eligibility requirements, are a "final destination" where a large proportion of older adults never arrive. Transitions to a nursing facility were most likely for older women with low levels of education and poor health which were the characteristics associated with mixed informal and the mixed publicly paid CN. This finding has been reported previously (Algera et al. 2004). However, other studies put this into perspective, as informal care delays institutionalization and leads to a (relatively) lower level of formal care (Van Houtven and Norton 2004). In addition, Carvalho et al. (2019) report gender differences in the point where older adults consider their disability too severe to live at home and be cared for by a spouse, with women tending to prefer institutionalization at lower levels of disability.

We demonstrated the robustness of the determinants of care use (Andersen and Newman 2005; Babitsch et al. 2012) in a longitudinal perspective. Our longitudinal design showed that need factors, including declining functional and cognitive limitations and the number of chronic diseases, appear to have the strongest influence on transitions. This is similar to findings from studies on care arrangements (Dostie and Léger 2005; Soldo et al. 1990; Witvorapong 2011). The use of CN typologies in a longitudinal setting was not only novel, but linking them to determinants provides information for policy makers to anticipate what support is needed to promote the right CNs. Loss of a spouse did not often occur at the time when participants transitioned to another network, which could be explained by the fact that the spouse was already unable to provide care in the year before death.

However, the methods we used were mainly descriptive. In addition, the determinants we examined were not theoretically exhaustive. We used longitudinal data with 3-year waves, which had the limitation that certain significant transitions that occurred over a shorter period of time may not have been captured. As the frailest older adults move to a care facility, this may have led to an underestimation of the number of older adults who move to a care facility and an overestimation of those who die without ever moving to a care facility. Future studies could include determinants such as social capital, mastery and attitudes towards care, income, and the type of formal and informal care available in the region (Blomgren et al. 2008; Broese van Groenou 2020; Jacobs et al. 2018).

## Conclusion

To learn more about the stability of CNs, we mapped transitions between CN types. We identified the following five types of CNs: (1) no care, (2) privately paid, (3) mixed informal, (4) mixed publicly paid, (5) co-resident. The co-resident network was the most unstable and had a high transition rate to nursing homes. The privately paid CN was moderately stable, with participants often transitioning to a mixed informal, but rarely to a mixed publicly paid CN. There were also moderately frequent transitions from the no-CN, with transitions to the privately paid CN being the most likely, but other transitions also occurred. The two mixed CNs were the most stable, and transitions to a care facility were most likely for these types. For all types of CNs, transitions appeared to be strongest induced by a deterioration in health.

### Supplementary Information

Below is the link to the electronic supplementary material.

Supplementary file1 (DOCX 23 kb)
