# Supplementary material for: Transitions between care networks: a prospective study among older adults in the Netherlands
Source: Eur J Ageing. 2024 Aug 13;21(1):22. doi: 10.1007/s10433-024-00817-x (PMC11322471; doi:10.1007/s10433-024-00817-x)
Supplement: Supplementary file 1 — Supplementary file1 (DOCX 23 kb) [file 10433_2024_817_MOESM1_ESM.docx]

For the model selection we tested which model was best fit at every wave by using LCA. In accordance with the three step model (Vermunt, 2010) we compared the models without covariates, and concluded which were the most appropriate number of classes per wave. Because co-modelling missingness should not change the number of defined classes (only the sizes of these classes) (Haviland et al, 2011), we did not model missing states in the LCA’s.

| Table S1. Model fit statistics for latent class analysis per wave | | | | | | | | | | |
| --- | --- | --- | --- | --- | --- | --- | --- | --- | --- | --- |
| Wave 1 | Bootrst LRT | | BIC | | Entropy | | Size smallest class | | N | |
| 1 |  | | 5756.570 | |  | |  | | 1413 | |
| 2 | 2856.525 | | 5576.701 | | 0.578 | | 403 | | 1413 | |
| 3 | -2741.203 | | 5587.563 | | 0.719 | | 53 | | 1413 | |
| 4 | -2721.247 | | 5629.228 | | 0.645 | | 45 | | 1413 | |
| 5 | -2716.692 | | 5673.253 | | 0.829 | | 10 | | 1413 | |
| Wave 2 |  |  | |  | |  | |  | |  |
| 1 |  |  | |  | |  | |  | |  |
| 2 | -2395.220 | 4697.681 | | 0.542 | | 344 | | 1095 | |  |
| 3 | -2303.350 | 4701.902 | | 0.750 | | 83 | | 1095 | |  |
| 4 | -2280.966 | 4743.544 | | 0.883 | | 35 | | 1095 | |  |
| 5 | -2277.292 | 4783.227 | | 0.896 | | 24 | | 1095 | |  |
| 6 | -2272.639 | 4827.518 | | 0.741 | | 15 | | 1095 | |  |
| 7 | -2270.261 | 4872.290 | | 0.735 | | 8 | | 1095 | |  |
| 8 | -2268.181 | 4916.991 | | 0.830 | | 6 | |  | |  |
| Wave 3 |  | |  | |  | |  | |  |  |
| 1 |  | | 4398.226 | |  | |  | | 872 |  |
| 2 | -2178.800 | | 4270.616 | | 0.643 | | 334 | | 872 |  |
| 3 | -2091.298 | | 4291.238 | | 0.713 | | 117 | | 872 |  |
| 4 | -2077.911 | | 4320.351 | | 0.770 | | 19 | | 872 |  |
| 5 | -2068.770 | | 4353.938 | | 0.735 | | 13 | | 872 |  |
| 6 | -2061.866 | | 4392.757 | | 0.804 | | 8 | | 872 |  |
| 7 | -2057.577 | | 4431.996 | | 0.822 | | 4 | | 872 |  |
| 8 | -2053.560 | | 4473.424 | | 0.909 | | 4 | | 872 |  |

Although the BIC was lowest for the two-class solution at every wave, we decided to choose the five class solution, because it relatively had the best entropy and a reasonably sized smallest class. In addition, it was most in accordance with what we expected based on previous studies.

Then we checked whether measurement invariance was applicable, by first checking whether the distributions and means of the dependent variables varied greatly across time (which was not the case), and then comparing the log-chi2-tests of a model with and a model without measurement invariance (for this comparison, we compared both the LTA without the missing and the LTA with the missingness indicators included).

Eventually we decided a model with five classes at each wave, and two forms of missing states (deceased, moved to a care facility).

| Table S2. Latent Transition Analysis (all with 2 classes for missing and time invariant) | | | |
| --- | --- | --- | --- |
| N of classes | Highest loglikelihood | BIC | AIC |
| 3 | -7693.406 | 15923.568 | 15534.811 |
| 4 | -7539.936 | 15812.472 | 15281.871 |
| 5 | -7432.365 | 15822.187 | 15128.729 |
| 6 |  |  |  |

References

Haviland, A.M., Jones, B.L. and Nagin, D.S. (2011) Group-based trajectory modeling extended to account for nonrandom participant attrition, *Sociological Methods and Research*, 40(2), 367–390, doi: 10.1177/0049124111400041.

Vermunt, J.K. (2010) Latent class modeling with covariates: Two improved three-step approaches, *Political Analysis*, 18(4), 450–469, doi: 10.1093/pan/mpq025.
